# *Shank2/3* double knockout-based screening of cortical subregions links the retrosplenial area to the loss of social memory in autism spectrum disorders

**DOI:** 10.1038/s41380-022-01756-8

**Published:** 2022-09-13

**Authors:** Débora Garrido, Stefania Beretta, Stefanie Grabrucker, Helen Friedericke Bauer, David Bayer, Carlo Sala, Chiara Verpelli, Francesco Roselli, Juergen Bockmann, Christian Proepper, Alberto Catanese, Tobias M. Boeckers

**Affiliations:** 1 Institute of Anatomy and Cell Biology, Ulm University, 89081 Ulm, Germany; 2 International Graduate School, Ulm University, 89081 Ulm, Germany; 3 German Center for Neurodegenerative Diseases (DZNE), Ulm site, 89081 Ulm, Germany; 4 Department of Neurology, Ulm University, 89081 Ulm, Germany; 5 CNR, Institute for Neuroscience, Milano, Italy

**Keywords:** Neuroscience, Autism spectrum disorders, Molecular biology

## Abstract

Members of the Shank protein family are master scaffolds of the postsynaptic architecture and mutations within the SHANK genes are causally associated with autism spectrum disorders (ASDs). We generated a Shank2-Shank3 double knockout mouse that is showing severe autism related core symptoms, as well as a broad spectrum of comorbidities. We exploited this animal model to identify cortical brain areas linked to specific autistic traits by locally deleting Shank2 and Shank3 simultaneously. Our screening of 10 cortical subregions revealed that a Shank2/3 deletion within the retrosplenial area severely impairs social memory, a core symptom of ASD. Notably, DREADD-mediated neuronal activation could rescue the social impairment triggered by Shank2/3 depletion. Data indicate that the retrosplenial area has to be added to the list of defined brain regions that contribute to the spectrum of behavioural alterations seen in ASDs.

## Introduction

Autism spectrum disorders (ASD) are a group of heterogeneous neurodevelopmental disorders characterized by deficits in social communication and social interaction, as well as restricted and repetitive behaviours. Additionally, individuals diagnosed with ASD often present cognitive impairments, language development delay/absence and a broad spectrum of comorbidities, including hyperactivity and anxiety disorders [1]. Despite recent genetic studies on ASD have pointed to a complex and heterogeneous aetiology [2], there is increasing evidence that ASD-related genes converge to common biological pathways that include chromatin modifiers, translational regulators, synapse formation/function and cortical development [3].

Disorganized cortical structure [4], altered cortical thickness [5], changes in cortical connectivity [6] and dendritic integration [7] are often observed in individuals affected by ASD. Given the importance of the cortex in cognition, sociability and sensory integration (processes commonly found to be affected in autism; [8]), such anatomical changes in cortical subregions are thought to underlie ASD-phenotypes [3, 9]. Interestingly, a neuroimaging study showed that increased cortical surface area between 6 and 12 months of age could predict ASD diagnosis. Moreover, this generalized cortical enlargement was linked to the development and severity of social deficits [10].

In line with the human studies, experiments utilizing ASD-mouse models have reported not only synaptic abnormalities, but also cortical dysfunction [9]. For example, abnormal activity has been observed in cortical regions as a result of loss of synaptic proteins such as Shanks [11, 12]. Shank1, Shank2 and Shank3 are scaffold proteins at the postsynaptic density (PSD) of excitatory synapses [13–15]. Here they interact directly or indirectly with scaffolding and signalling proteins, in order to regulate synaptic development, structure and function [16]. Abnormalities within the three *SHANK* genes are detected in the whole spectrum of autism and a higher cognitive impairment is observed when mutations are present in the *SHANK3* and *SHANK2* genes compared to *SHANK1* [17]. In mice, Shank3 deficiency in the prefrontal cortex [18, 19] and anterior cingulate cortex [20], two cortical subregions commonly found altered in ASD patients [9], induces social deficits. On the other hand, *Shank3* loss in the neocortex results in increased repetitive behaviours [21].

Despite the significant advances in identifying brain regions that are responsible for typical behavioural alteration in autism, the anatomical basis underlying specific ASD-phenotypes are still not clear. Given the importance of the cerebral cortex in the manifestation of autistic-like behaviours, we exploited a novel screening method based on the local deletion of *Shank2* and *Shank3* in 10 different cortical areas of double floxed mice. We found that the local reduction of these two postsynaptic proteins in the retrosplenial area (RSP) is sufficient to induce social memory deficits, without affecting other behavioural phenotypes. Interestingly, the sudden loss of Shank2 and Shank3 proteins after physiological neuronal development [22] resulted in a drastic local reduction of excitatory synapses, which was not rescued by the acute increase in neuronal activity.

## Materials and methods

### Mice

Mice were housed in mixed-genotype groups (2–4 per cage) and randomly selected for behavioural or biochemical experiments. Cages were kept in individual cage ventilation systems, at constant temperature and humidity, under a 12 h light/dark cycle (lights on at 6 am). Food and water were available *ad libitum*.

*Shank2-Shank3* double knockout mouse (dKO) were generated by crossing the *Shank2Δ7*^−/−^ with the *Shank3Δ11*^−/−^ single KO model [23] to obtain double heterozygous mice that were crossed in order to generate *Shank2*^−/−^*-Shank3*^−/−^ dKO.

Conditional gene knockout targeting vectors for *Shank2 and Shank3* were designed by flanking the exon 7 and the exon 11, respectively, with a loxP site on one side and frt - neomycin gene - frt - loxP site on the other side. The linearized targeting constructs were electroporated into recombinant inbred embryonic stem cells (RI-ES cells) and the correctly targeted cells (proved by Southern blotting) were implemented in C57BL/6 blastocysts. Then, the frt - neomycin - frt cassette was deleted by pronuclear microinjection of the FLP recombinase. The blastocysts were transferred into pseudopregnant C57BL/6 females using standard procedures. Germline transmission was tested by polymerase chain reaction (PCR) using the following primers: *Shank2*^*fx/fx*^ for WT allele forward TCCGCAGACCACTTTATTCC, for WT allele reverse GGGTGTGAATTCCTCAATGG; for floxed allele forward TCCGCAGACCACTTTATTCC, for floxed allele reverse AAGAAGCCCCAGAAGTGACA; *Shank3*^*fx/fx*^ for WT allele forward GTCTCTGTGGTTGGGGTGTC, for WT allele reverse CAGTGGAGCAAGCCACATTA, for floxed allele forward CCTCTAGGCCTGCTAGCTGTT, for floxed allele reverse AAGAAGCCCCAGAAGTGACA.

Homozygous conditional knockout mice for both *Shank2* and *Shank3* were generated by crossing the *Shank2*^*fx/fx*^ with the *Shank3*^*fx/fx*^ single mutant mice to generate *Shank2*^*fx/fx*^-*Shank3*^*fx/fx*^ mice (dKO^fx/fx^).

Subsequent dKO and dKO^fx/fx^ mouse genotyping was determined by PCR using the above primers. WT animals were purchased from Charles River Laboratories. All mice had a pure C57BL/6 J background.

Animal experiments were carried out in compliance with the guidelines of the Italian Ministry of Health, Federal Government of Germany and the local ethics committee (Ulm University; ID Number: 1360).

### Behavioural experiments

Male and female dKO mice, 8–12 weeks old, were used for behavioural experiments. Behavioural experiments with male dKO^fx/fx^ mice were conducted 6 weeks post-injections (≥ 11 weeks old). Mice were habituated to the behaviour room at least 1 h before behavioural experiments. All the tests were performed between 8 am and 5 pm (light phase of the mouse light/dark cycle), except for the nest building test. Behavioural experiments were performed by experimenters blinded to the genotypes and groups. To allocate mice into experimental groups, a stratified randomization method was used.

#### Nest building test

Mice were individually housed in a new home cage with fresh bedding and without environmental enrichment items. Then, a single nestlet was added 1 h before the dark phase. Nest quality was assessed the following morning according to a 5-point rating scale [24].

#### Marble burying test

The marble burying test was performed in a soundproof anechoic room under dim red light (10 lux), in order to ensure that tested mice are calm and stress-free during the test. After 15 min of habituation, the mouse was placed in a clean cage (26.5 cm length x 20 cm width x 14 cm height) containing 4 cm of fresh bedding and 12 marbles placed in a regular pattern on the surface. After 30 min, the number of marbles buried (to 2/3 their depth) with bedding was quantified [25].

#### Three-chamber paradigm test

Social approach behaviour and preference for social novelty was performed as previously described [26]. Briefly, the arena was divided in three equal chambers: middle chamber and another two chambers where the wire cages for the stranger mice were placed. The test consisted of three-10 min trials: [1] habituation, [2] sociability and [3] social novelty.

1. The subject mouse was allowed to freely explore the whole setting and the 2 empty stimulus cages in each side-chamber.
2. The tested mouse was gently guided to the middle chamber and an unfamiliar mouse (stranger 1 – S1) was introduced to one of the stimulus cages. Subsequently, the tested mouse was left to explore all chambers.
3. After the subject mouse was confined to the middle chamber, a second unfamiliar mouse (stranger 2 – S2) was placed in the other stimulus cage. Finally, the tested mouse was allowed to explore the whole apparatus again.

The time spent in each chamber, as well as, the time in close proximity to the wire cages (time spent sniffing) was analysed using the tracking software EthoVision XT (Noldus, Wageningen, Netherlands). Social preference index was calculated as (S1 − E) / (S1 + E) and social novelty preference index as (S2 − S1) / (S1 + S2), where S1 is the time spent in close proximity with the stranger 1, S2 is the time spent in close proximity with the stranger 2 and E is the time spent in close proximity with the empty cage. The position of the empty cage and the cage with S1 were alternated among tests. C57BL/6JRj mice of the same sex and age of the tested mice were used as strangers and were previously habituated to the wire cages.

#### Female urinary pheromone-elicited ultrasonic vocalization

Ultrasonic vocalizations (USVs) of male mice were recorded in the home cage under dim red light (9 lux) in a soundproof chamber as previously described [26]. After 15 min habituation, 20 μl fresh urine (1:1 diluted in ddH_2_O) of an unfamiliar C57BL/6JRj female (age 9–10 weeks) was presented on a cotton swab to the tested mouse. The oestrous phase of the female was confirmed by vaginal smear evaluation [27].

USVs were recorded for a period of 3 min using a condenser ultrasound microphone (CM16, Avisoft Bioacoustic, Berlin, Germany) sensitive to frequencies of 10–180 kHz. The microphone was connected to a computer with Avisoft recorder Software (UltraSoundGate 116 USB, version 3.2, Avisoft Bioacoustics, Berlin, Germany).

Starting-point of the call-analysis was determined by a digital control-signal, which was sent to a LED light by the sound recording system, mounted in the field of view of the camera. Video recordings were played back (frame by frame) using Ulead VideoStudio software (version 7.0, Corel Corporation, Ottawa, Canada). The starting-point of the analysis was defined by turning on the LED light.

Analysis of audio recordings: WAV file were transferred to SASLab pro Software (version 4.5, Avisoft Bioacoustics, Berlin, Germany) and a Fast Fourier transform (FFT) was performed (512 FFT length, 100% frame, Hamming window, time resolution: 75% overlap). The spectrogram was created at a frequency resolution of 586 Hz and a time resolution of 0.4267 ms. Background noise was removed from the spectrogram by cutting off frequencies lower than 15 kHz.

#### Ultrasonic vocalizations during male-female interaction

After 15 min habituation period in a soundproof chamber, an unfamiliar C57BL/6JRj female in oestrus (confirmed through vaginal smear) was introduced in the home cage of the male subject mice for a period of 3 min. USVs were recorded with the same settings and equipment as described above. Starting-point of the social interaction and call analysis was defined by the female mouse having all four paws on the bottom of the cage.

All males had previous experience (4 days before the test) to mature females (8-9 weeks) for a period of 3 min. The test session was video-recorded and subsequently scored frame by frame for social behavioural analysis using Ulead VideoStudio software version (25/s; version 7.0, Corel Corporation, Ottawa, Canada). Time in contact was calculated as cumulative time spent (2 ≤ cm) in: anogenital sniff (sniffing of the partners anogenital region), nose to nose sniff (sniffing the snout or head region of the partner), body sniffing (sniffing any body region, with exception of head and anogenital region), mounting (any attempt and act of mount), following (walking straight behind the partner or in parallel line with the partner), push – crawl (crawling over or under the partner from one side to the other), pushing past (squeezing between the cage wall and the partner). Number of attacks were defined as attacks carried out over the dorsal side or front side of the female mouse.

#### Buried food test

Buried food test was performed as previously reported [28] with minor modifications. To prevent neophobia, 4 consecutive days prior to the olfactory test, 3–4 pieces of food high in carbohydrates (Froot Loops, Kellogg’s, Battle Creek, Michigan, US) were placed in the home cage of subject mice. Froot Loops-consumption was controlled in the following morning. Only mice, which consumed the Froot Loops pieces were included in this test. 20 h before the test, mice were food-deprived with water available *ad libitum*. On the test day, mice were habituated to a clean test cage, containing a 3 cm layer of bedding for a period of 10 min. Next, mice were transferred briefly into a clean cage and 4 Froot Loops were buried 3 cm below the bedding of the test cage. The subjected mice were then returned to the cage for a 5 min test session. The latency for the mouse to find the buried food (time until the mouse uncovered the food and holding it with their forepaws) was video-recorded.

### Repetitive behaviours

Stereotypic behaviours were measured in a soundproof chamber under dim red light (8 lux) as previously described [26] with minor modifications. In brief, each mouse was individually placed in an empty standard mouse cage filled with a thin layer of fresh bedding. After a habituation period of 15 min, mice were video-recorded for 10 min and the video was subsequently scored frame by frame for repetitive behaviours (self-grooming, jumping and upright scrabbling) using Ulead VideoStudio software version (25/s; version 7.0, Corel Corporation, Ottawa, Canada). The measured grooming behaviours included scratching of face, head or body with the two forelimbs, or licking body parts. Jumping was defined when the mouse was standing on its hind legs either at the corner of the cage or along the side-walls, and jumped so that both hind legs were simultaneously off the ground. Upright scrabbling was scored when the test mouse was standing in an upright position and tried to climb up against the cage wall with the two hind legs alternatively touching the ground. Time spent self-grooming, jumping and upright scrabbling is presented in percentage (%) out of total measured time (10 min).

#### Open field

The open field test was performed as previously reported [26]. Briefly, the open field chamber consisted of an opaque plexiglas arena, which was homogeneously illuminated at 100 lux. Mice were carefully introduced in the centre of the open field and allowed to freely explore the arena (50 × 50 cm) with a (20 × 20 cm) centre zone for a period of 30 min. Time spent in the centre zone and distance travelled were quantified using Viewer 2 software (Bioserve, Bonn, Germany).

#### Elevated plus maze

The elevated-plus maze test was conducted as previously described [26]. The maze was positioned 60 cm above the floor and consisted of two open and two enclosed arms (with 16 cm high walls) that extended from a central platform (5 × 5 cm). The light intensity in the open arms was 100 lux and in the closed arms was 40 lux. Mice were introduced in the central platform, facing one of the enclosed arms and allowed to explore the maze for 10 min. Time spent in open arms and distance travelled was analysed using Viewer 2 software (Bioserve, Bonn, Germany).

#### Y-maze

Spontaneous alternation, a measure of spatial working memory, was analysed in a symmetrical Y-maze (three arms, 40 × 9 cm with 16 cm high walls). Each animal was allowed to freely explore the maze for 5 min and arms choices (all four paws entering one arm) were scored. Alternation was determined by recording the order of the visited arms (arm A, arm B or arm C). Overlapping triplets of three arm visits was counted as one complete spontaneous alternation. To calculate the percentage of alternation, the following formula was used: % Alternation = (Number of Alternations / [Total number of arm entries - 2]) x 100 [29]. The Y maze arena was carefully cleaned with 70% ethanol between each animal to remove odour cues. Videos were recorded and analysed using Viewer 2 software (Bioserve, Bonn, Germany).

#### Olfactory habituation/dishabituation test

Odours presentation was performed as previously described [28] with minor changes. In brief, tested mice were first habituated for 30 min to a new and clean cage containing a thin layer of bedding and an empty embedding cassette macrosette (EE16.1, Carl Roth, Karlsruhe, Germany) on the grid bars of the empty stainless steel food hopper. Afterwards, 10 μL of non-social (ddH_2_O) and social odours (male urine or female urine) were presented on a piece of clean filter paper (2 × 2 cm, Whatman no. 5, GE Healthcare, Chicago, Illinois, US), which was placed inside of the empty embedding cassette macrosette. Odours were presented in three consecutive trials for a duration of 2 min (inter-trial interval: 1 min) in the following order: water, water, water; male urine, male urine, male urine; female urine, female urine, female urine. Urine was collected from five males or females (4 months old, C57BL/6JRj). The time spent sniffing the embedding cassette macrosette was recorded and subsequently quantified.

#### Barnes maze

The Barnes maze test was performed as previously reported [30] with minor modifications. Briefly, the paradigm consisted in a grey circular platform (100 cm diameter), elevated 60 cm from the floor, with twenty closed holes (5 cm diameter) evenly spaced around the circumference. An escape box (target) was placed under one of the holes. On the pre-training trial, the tested mouse was placed in the middle of the maze inside of an opaque chamber, and light sources were turned on (600 lux). After 10 s, the opaque chamber was lifted, and the mouse was gently guided to the escape box, where it remained for 2 min. Subsequently, the first training trial started. At the beginning of each trial, the tested mouse was placed in the opaque chamber, and 10 s after turning on the lights, the mouse was allowed to freely explore the apparatus. The training trial finished when the mouse entered the escape box or after 3 min have elapsed. Mice were allowed to stay inside the escape box for 1 min. Animals were trained to locate the escape box during four training trials per day/4 days. Training trials were separated by 15 min. The escape box position was different among tested mice and its spatial location with respect to visual extra-maze cues was consistent between trials. Moreover, the apparatus was rotated every day to eliminate the use of intra-maze cues. 24 h after the last training trial, a 90 s probe trial was conducted without the escape box to assess spatial memory. Trials were recorded and analysed using the tracking software EthoVision XT (Noldus, Wageningen, Netherlands).

### Viral vectors

pAAV.CMV.PI.EGFP.WPRE.bGH (AAV9-CMV-GFP) was a gift from James M. Wilson (Addgene viral prep #105530-AAV9; http://n2t.net/addgene:105530; RRID:Addgene_105530 - Addgene, Watertown, Massachusetts, US). pAAV.CMV.HI.eGFP-Cre.WPRE.SV40 (AAV9-CMV-GFP-Cre) was a gift from James M. Wilson (Addgene viral prep #105545-AAV9; http://n2t.net/addgene:105545; RRID:Addgene_105545). pAAV-hSyn-DIO-hM3D(Gq)-mCherry [AAV9-hSyn-DIO-hM3D(Gq)-mCherry] was a gift from Bryan Roth (Addgene viral prep #44361-AAV9; http://n2t.net/addgene:44361; RRID:Addgene 44361).

The viral titer was (1.9–5) × 10^13^ particles per ml. All viral vectors were aliquoted and stored at −80 °C until use.

### Intracerebral injections

Stereotactic injections of adeno-associated virus (AAVs) were performed at postnatal day (P)34–36, as previously described [31] with minor changes. In brief, WT or dKO^fx/fx^ mice were pre-treated with buprenorphine (0.1 mg/kg) and meloxicam (1 mg/kg) 30 min before being anesthetized with 5% sevoflurane. Afterwards, the mice were fixed in a stereotaxic frame (model 962, David Kopf Instruments, Tujunga, California, US) and kept under continuous anaesthesia with 2–3% sevoflurane. Body temperature was monitored by a rectal probe and maintained at 37 °C. Then, scalp was incised to expose the skull and the burr holes were drilled using a steel bur round with 0.5 mm of diameter. All skull measurements were made relative to bregma and the coordinates for each region (Supplementary Table 1) were defined based on “The Mouse Brain In Stereotaxic Coordinates” [32]. For the cortical screening, 10 coordinates were defined based on a grid designed on the anterior-posterior and medial-lateral axes (AP from −3 mm to + 3 mm / ML from −3 mm to + 3 mm) of the mouse brain atlas (Fig. 3A and Supplementary Table 1). The dorsal-ventral (DV) coordinates were defined in order to hit all the cortical layers of each region.

For the conditional knockout experiments in the nucleus accumbens (ACB), olfactory areas (OLF) and retrosplenial area (RSP), 200–300 nl of AAV9-CMV-GFP or AAV9-CMV-GFP-Cre was bilaterally injected using a pulled-glass capillary. For the cortical screening experiments, 200–300 nl of AAV9-CMV-GFP-Cre was bilaterally injected into each coordinate. For the DREADD experiments, a 200–300 nl mixture containing equal amounts of AAV9-CMV-GFP and AAV9-hSyn-DIO-hM3D(Gq)-mCherry or AAV9-CMV-GFP-Cre and AAV9-hSyn-DIO-hM3D(Gq)-mCherry was bilaterally injected into the RSP. Viruses were delivered at a rate of ~ 50 nl/min using a Picospritzer microfluidic device (Parker, Hollis, New Hampshire, US). Following viral injection, the capillary was held at the site for 10 min to prevent backflow of the virus.

### Chemogenetics agonist administration

Clozapin-N-Oxid (CNO, Sigma-Aldrich, Burlington, Massachusetts, US) was dissolved in dimethyl sulfoxide (DMSO, Sigma-Aldrich) and administered intraperitoneally at the dose of 5 mg/kg in saline. Animals received either a single or 5 daily doses of CNO. The last administration was always performed 30 min before the beginning of the three-chamber paradigm test. Control animals were treated with vehicle.

### Primary antibodies list

Rb anti-Shank2 (“ppI-SAM pab SA5192”, 1:1000 in WB and 1:500 in IHC) and Rb anti-Shank3 (“PRC pab”, 1:1000 in WB and 1:500 in IHC) were previously characterized [13, 23]. Ms anti-Actin (A2228, Sigma-Aldrich, Burlington, Massachusetts, US, 1:250000 in WB); Rb anti-Homer 1b/c (160 022, Synaptic Systems, Goettingen, Germany, 1:500 in IHC); Gp anti-VGLUT 1 (135 304, Synaptic Systems, 1:500 in IHC); Gp anti-NeuN (266 004, Synaptic Systems, 1:500 in IHC); Rb anti-c-Fos [2H2] (ab190289, abcam, Cambridge, UK, 1:500 in IHC); Ck anti-VGAT (131 006, Synaptic Systems, Goettingen, Germany, 1:500 in IHC).

### Western blot

Western blot of brain tissue was performed as previously reported [23]. In brief, cerebral cortex either of WT or dKO mice were homogenized in HEPES-buffered sucrose (320 mM sucrose, 5 mM HEPES, pH 7.4) containing protease inhibitors. Equal amounts of 10 µg protein were separated by SDS-PAGE and subsequently blotted on nitrocellulose membranes according to standard protocols. Membranes were blocked with 5% skim milk powder (Sigma-Aldrich, Burlington, Massachusetts, US) in Tris-buffered saline with 0.1% TWEEN-20 (TBST). After overnight incubation with primary antibodies, the membranes were washed 3 times with 0.1% TBST and incubated with HRP-conjugated secondary antibodies (Dako, Agilent Technologies, Santa Clara, California, US) for 1 h. Membranes were then washed 3 times with 0.1% TBST and immunoreactivity was visualized on X-ray film (GE Healthcare, Chicago, Illinois, US) using the SuperSignal detection system (Thermo Scientific, Waltham, Massachusetts, US).

### Brain sectioning and immunohistochemistry

Brain sectioning and immunohistochemistry were performed as previously described [33], with minor changes. 1 hour after behavioural experiments, dKO^fx/fx^ mice were transcardially perfused with 25 mL ice-cold phosphate buffered saline (PBS, Gibco, Waltham, Massachusetts, US) followed by 50 mL ice-cold 4% paraformaldehyde (PFA, Sigma-Aldrich, Burlington, Massachusetts, US) in PBS (pH 7.4). Afterwards, brains were incubated in 4% PFA overnight at 4 °C and then, cryoprotected in 30% sucrose in PBS for 48 h at 4 °C. Finally, samples were snap-frozen in optimal cutting temperature compound and serially sectioned using a Leica CM3050 S cryostat (Leica Biosystems, Wetzlar, Germany) into 40 μm coronal sections.

Free-floating brain coronal sections were blocked in 3% bovine serum albumin (neoFroxx, Einhausen, Germany) and 0.3% Triton (Roche, Basel, Switzerland) in PBS for 2 h at room temperature (RT). Slices were thereafter incubated with the primary antibody diluted in blocking solution for 48 h at 4 °C. After 3 washes in PBS, brain sections were incubated with the secondary antibody coupled to Alexa Fluor 568, 647 or AMCA (Jackson ImmunoResearch, Cambridge, UK, 1:500) in blocking solution for 2 h at RT. After 3 further washes in PBS, the sections were mounted with ProLong Gold Antifade Mountant either with or without DAPI (Invitrogen, Waltham, Massachusetts, US).

### Image acquisition and analysis

Volumetric measurements of single areas targeted in the cortical screening were performed as previously described [31]. In brief, tile-scan images of brain coronal sections were acquired using a DMI6000B slide-scanning microscope (Leica Microsystems, Wetzlar, Germany) equipped with a 5x/0.12 objective at 16-bit depth. Afterwards, GFP-Cre-positive brain regions were manually registered and individually cropped out of each image, using structural landmarks (such as shape of corpus callosum and expansion of ventricles) accordingly to the Allen Brain Anatomical Reference Atlas [34]. Volume measurements were performed by calculating the area of scaled and cropped images and multiplying it by the thickness of the section (40 μm), using a custom-made macro in Fiji [35]. Single GFP-Cre positive areas which count for less than 10% of the total injection volume were grouped and named as adjacent areas. Due to the uncertainty in defining borders and in agreement with functional similarity, anterolateral visual area, anteromedial visual area, posteromedial visual area and primary visual area were grouped and named as visual areas (VIS). Likewise, accessory olfactory bulb, anterior olfactory nucleus, dorsal peduncular area, main olfactory bulb, piriform area and taenia tecta were grouped and nominated as olfactory areas (OLF).

Tile scan images of coronal and sagittal brain slices immunostained with antibodies against Shank2 and Shank3 proteins were acquired with the DMI6000B slide-scanning microscope using the 5x/0.12 objective. For the analysis of Shank2 and Shank3 intensity mean within the prefrontal cortex (PFC), secondary motor area (MOs), anterior cingulate area (ACA) and RSP, the Fiji software [35] was used. Analysis was carried out on 3 WT mice and 3 sagittal slices were used per animal.

Images of GFP- and GFP-Cre-expressing brain areas immunostained for pre- and postsynaptic markers were acquired using a DMi8 laser-scanning microscope (Leica Microsystems) equipped with an ACS APO 63x oil immersion objective with a resolution of 2048 × 2048 pixels. The acquisition depth in the z-axis was 2.2 μm at acquisition intervals of 0.22 μm. Analysis of puncta number was conducted using Imaris software (Bitplane, Zürich, Switzerland) as previously described [36]. In brief, puncta from three randomly selected regions of interest (ROI, 20 μm x 20 μm) were identified using the “intensity mean” parameter. Then, to quantify the colocalization between Homer1b/c and VGlut1 the “colocalize spots” tool was used.

For the quantification of neurons-positive for Cre, Cre/Gq, as well as c-Fos/Cre/Gq, confocal images were acquired with a resolution of 512 × 512 pixels using an ACS APO 40x oil immersion objective. The acquisition depth in the z-axis was 13.8 μm at acquisition intervals of 0.6 μm. NeuN signal was used to guarantee the identification of neuronal nuclei. The confocal images were then imported in LAS AF Lite software (Leica Microsystems) and the total number of neurons-positive for Cre, Cre/Gq, c-Fos/Cre/Gq, was manually quantified.

Immunohistochemistry analysis was carried out on at least 3 mice. 3 coronal slices per animal were stained with each antibody/antibody combination and 3 confocal images were acquired per injection site.

### Statistical analysis

Statistical analyses were performed using Chi-square test, unpaired t-test with Welch’s correction, Mann–Whitney test, one-way ANOVA followed by Tukey’s multiple comparisons test, Kruskal–Wallis test followed by Dunn’s multiple comparisons test or two-way ANOVA followed by Sidak’s multiple comparisons test. The preference index for the three-chambers test in chemogenetic experiments was calculated using paired t-test. Sample normality was tested using the Shapiro-Wilk test. Synapse-related immunostaining data are presented as fold change relative to the control. Statistical analyses were performed using Prism (version 8.0.1, GraphPad, San Diego, California, US). Significance was set at *p* < 0.05 and displayed as follows: *p < 0.05; **p < 0.01;

***p < 0.001. All the results are displayed as mean ± standard deviation (SD). Number of animals used is indicated in the figure legends and detailed statistical information can be found in Supplementary Table 2.

## Results

### *Shank2/3* dKO mice show severe autistic-like behaviours

**Fig. 1:**
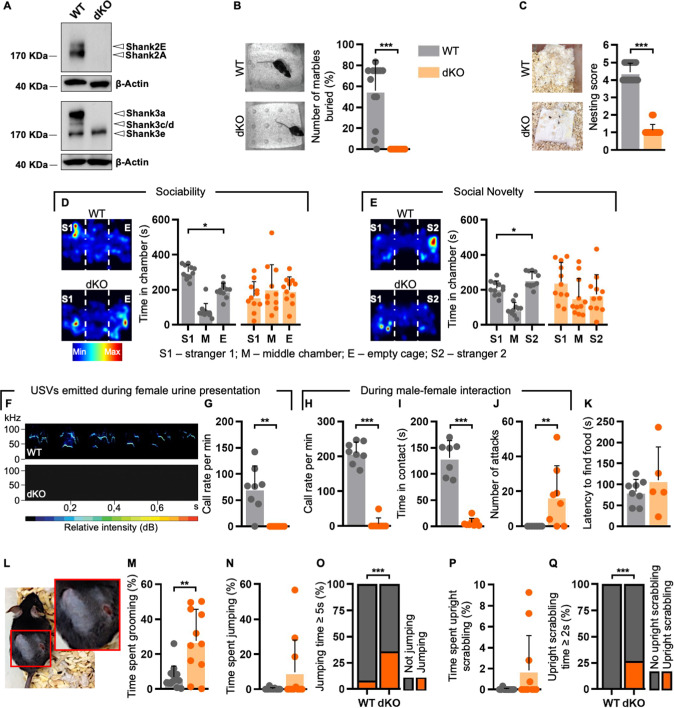
*Shank2/3* dKO mice show severe autistic-like behaviours. **A** Representative immunoblot showing the loss of Shank2A/E and Shank3a/c/d/e isoforms in cortical homogenates of dKO mice. **B** dKO did not bury any marble in comparison to WT mice; WT = 13, dKO = 10. **C** Impaired nest building in dKO mice; WT *n* = 16, KO *n* = 16. **D** Representative heatmaps of the three-chamber sociability trial (left) and dKO mice showed social deficits (right); WT = 11, dKO = 11. **E** Representative heatmaps of the three-chamber social novelty trial (left) and dKO mice displayed social memory deficits (right); WT = 11, dKO = 11. **F** Frequency spectrogram of typical ultrasonic vocalizations (USVs) emitted by WT and dKO male mice during female urine presentation. **G**, **H** dKO male mice emitted reduced USVs in the presence of female urine (**G**), as well as during direct interaction with a female (**H**); WT = 8, dKO = 8. **I**, **J** dKO mice showed social interaction deficits (**I**) and increased aggressive acts (**J**); WT = 7, dKO = 8. **K** dKO mice detected odour cues; WT = 8, dKO = 5. **L**, **M** dKO mice displayed skin lesions (**L**) due to increased self-grooming (**M**); WT *n* = 14, dKO *n* = 11. **N**–**Q** A significant number of dKO mice showed increased time spent jumping (**N**, **O**) and upright scrabbling (**P**, **Q**); WT *n* = 12, dKO *n* = 11 (**N**, **O**); WT *n* = 14, dKO *n* = 11 (**P**, **Q**). See Materials and Methods, as well as Supplementary Table 2 for detailed statistical analysis.

To analyse the impact of synaptic disruption on the manifestation of ASD-related traits, we generated a *Shank2-Shank3* double knockout mouse line (henceforth dKO) by crossing the *Shank2Δ7*^−/−^ with the *Shank3Δ11*^−/−^ single KO model [23]. This resulted in the loss of Shank2A/E and Shank3a/c/d major isoforms ([23, 37]; Fig. 1A).

First, we observed that dKO mice did not bury any marble in comparison to WT during the marble burying test, which was indicative of neophobia (11; Fig. 1B). In addition, we found that mutant animals were characterized by a strongly altered nest building behaviour (Fig. 1C). Since these data suggested that the innate sociability of dKO mice might be dramatically impaired, we further investigated the presence of specific behavioural characteristics typical of ASD in our dKO animals such as impaired social interaction and communication [1]. By performing the three-chamber paradigm test, we readily verified that dKO animals had no preference between a stranger mouse (S1) and an empty cage, while WT mice spent more time in the chamber containing the S1 animal (Fig. 1D and Supplementary Fig. 1A). Accordingly, the preference index for sociability was significantly reduced in dKO mice when compared to WT ones (Supplementary Fig. 1B). When the empty cage was replaced by a second stranger mouse (S2), control mice spent more time exploring the S2, whereas dKOs showed no preference between S1 and S2 (Fig. 1E and Supplementary Fig. 1C). Accordingly, a large number of dKO mice showed a strongly reduced preference index for the novel stimulus (Supplementary Fig. 1D).

In addition to the impaired social interaction, dKO mice displayed severe deficits in communication: indeed, none of the dKO male mice emitted ultrasonic vocalizations (USVs) after presentation of fresh urine from a female WT mouse on a cotton swab (Fig. 1F, G). Since previous studies have shown that male mice tend to emit more USVs during direct contact with females [38], we then tested whether dKO mice would emit calls in such a high social arousal situation. To this end, we introduced a female in the home cage of male WT and dKO mice and recorded the USVs emitted during 3 min of test. While WT animals emitted a high number of USVs and interacted with the female, dKO mice did not show any social interest toward the unfamiliar animal (Fig. 1H, I and Supplementary Video 1). In contrast, mutant mice reacted aggressively to the presence of the new mouse, by repetitively attacking the female during the test (Fig. 1J). To exclude the possibility that the impaired social behaviour observed in dKO animals might originate from altered olfactory functions, we performed a buried food finding test. No significant difference was detected in latency to find food between WT and dKO mice (Fig. 1K), indicating that mutant mice are able to detect odour cues.

Next, we analysed repetitive behaviours, another core symptom of ASD [1]. In comparison to WT, mutant mice spent significantly more time self-grooming, which in some cases reached an extent comparable to self-harming, as evidenced by the pronounced skin lesions observed on dKO animals (Fig. 1L, M). Additionally, a significant percentage of mutant mice showed repetitive jumping and upright scrabbling on the cage wall (Fig. 1N–Q and Supplementary Video 2).

To test anxiety and hyperactivity related behaviours, both ASD comorbidities [1], we performed the open field and the elevated plus maze paradigm tests. In the open field test, the time spent in the centre zone was not significantly different between WT and dKO mice (Supplementary Fig. 1E, F), suggesting no anxiety-like behaviour. However, the distance travelled by the dKO mice in the arena was significantly increased in comparison to the WT mice (Supplementary Fig. 1G), suggesting that dKO animals are hyperactive. In line with the open field test, WT and dKO mice spent similar time exploring the open arms of the elevated plus maze, but the distance travelled during exploration was higher in mutant mice (Supplementary Fig 1H–J). No cognitive impairments were detected in dKO mice, as the spontaneous alternation behaviour between WT and dKO mice in the Y-maze arena was not significantly different (Supplementary Fig. 1K).

### Loss of Shank2/3 proteins in the nucleus accumbens leads to repetitive behaviours

Since the loss of the two major PSD scaffolds induced a wide set of autistic-like alterations, we generated a conditional *Shank2-Shank3* double knockout mouse line (dKO^fx/fx^) in order to dissect the region-specific contribution to the development of the observed ASD symptoms. At first, we focused on the nucleus accumbens (ACB), a brain region previously associated with stereotypical behaviours [39] and social deficits [40] in different mouse models of autism.

**Fig. 2:**
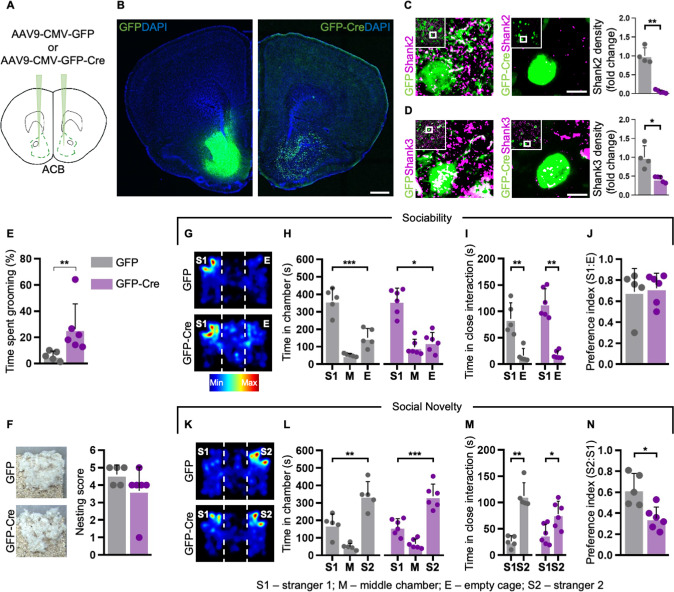
*Shank2/3* loss in the nucleus accumbens leads to repetitive behaviours. **A** Schematic of bilateral injections of AAV9-CMV-GFP or AAV9-CMV-GFP-Cre into nucleus accumbens (ACB) of dKO^fx/fx^. **B** Representative images showing GFP (left) and GFP-Cre (right) expression in the ACB. **C**, **D** Immunohistochemistry staining of the ACB using the Shank2 (**C**) and Shank3 (**D**) markers; GFP *n* = 4, GFP-Cre *n* = 5. **E** Cre-expressing mice displayed repetitive behaviours measured as time spent grooming; GFP *n* = 5, GFP-Cre *n* = 6. **F** Nest building behaviour; GFP *n* = 5, GFP-Cre *n* = 6. **G** Representative heatmaps of the three-chamber sociability trial. **H**–**J** GFP- and Cre-expressing mice spent more time exploring the S1 instead of the empty cage; GFP *n* = 5, GFP-Cre *n* = 6. **K** Representative heatmaps of the three-chamber social novelty trial. **L**–**N** GFP- and Cre-expressing mice spent more time together with the S2 instead of the S1 (**L**, **M**), but Cre-expressing mice displayed reduced preference for the novel stimulus in comparison to control animals (**N**); GFP *n* = 5, GFP-Cre *n* = 6. Scale bars: 500 μm in (**B**) and 5 μm in (**C**, **D**). See Materials and Methods, as well as Supplementary Table 2 for detailed statistical analysis.

At postnatal day (P)34-36, we bilaterally injected an adeno-associated virus (AAV)9-CMV-GFP-Cre (or an AAV9-CMV-GFP as control; Fig. 2A, B) in the ACB of dKO^fx/fx^ mice to locally abolish *Shank2* and *Shank3* expression. By assessing the efficacy of our approach of region-specific deletion, we confirmed that animals expressing Cre-recombinase showed a significantly lower density of Shank2 (Fig. 2C) and Shank3 (Fig. 2D) puncta in the ACB in comparison to the GFP control virus.

To determine the behavioural consequences of the local *Shank2/3* loss within the ACB we performed 6 weeks post-injections a set of behavioural experiments matching those performed with constitutive double KO animals. In agreement with what was observed in the dKOs, we noticed that Cre-injected mice spent more time self-grooming than control ones (Fig. 2E). In contrast, no significant changes were observed between control and *Shank2/3*-lacking animals in the nest building test (Fig. 2F). Interestingly, selective deletion of the two PSD scaffolds in the ACB did not have any effect on sociability: in the three-chamber test, GFP- and Cre-expressing mice showed indeed a significant and comparable preference for S1 compared to the empty cage (Fig. 2G–J), indicating that other regions might be responsible for the altered sociability observed in dKO mice. In addition, GFP- and Cre-expressing mice interacted with a novel social partner when the empty cage was replaced by a novel S2 animal (Fig. 2K–M). Nevertheless, the preference index for the novel stimulus was significantly lower in Cre-injected mice than in GFP-expressing controls (Fig. 2N), suggesting a contribution of the ACB to the formation of social memory.

### Cortical screening by local injection of Cre-expressing virus

**Fig. 3:**
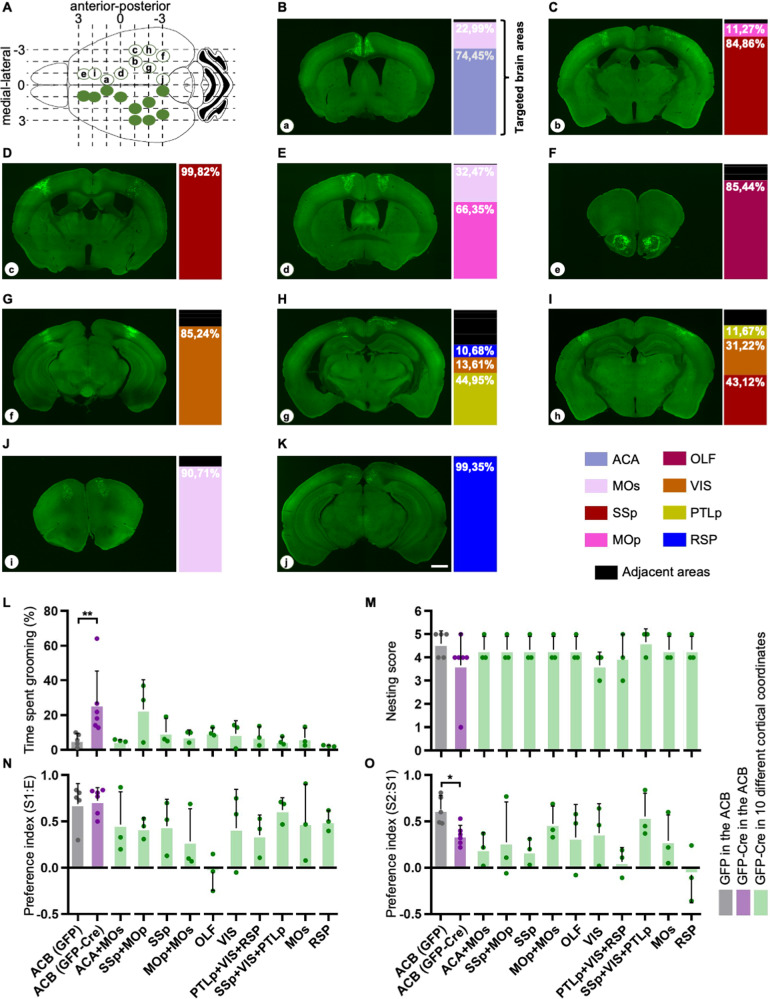
Cortical screening. **A** Schematic of bilateral injections of AAV9-CMV-GFP-Cre into 10 cortical coordinates (coordinate a-j). **B**–**K** Representative images showing GFP-Cre expression (left) and volumetric measurements of targeted single brain areas (right). ACA Anterior cingulate area; MOs, secondary motor area; SSp Primary somatosensory area, MOp Primary motor area, OLF Olfactory areas, VIS Visual areas, PTLp Posterior parietal association areas, RSP Retrosplenial area. **L**–**O** Time spent self-grooming (**L**), nesting score (**M**), preference index for sociability (**N**) and preference index for the novel stimulus (**O**) of dKO^fx/fx^ mice injected with AAV9-CMV-GFP-Cre in the different cortical coordinates. Nucleus accumbens (ACB) was used as a reference brain area for the screening; ACB (GFP) *n* = 5, ACB (GFP-Cre) *n* = 6, screening (GFP-Cre) *n* = 3 per each coordinate. Scale bar: 1 mm. See Materials and Methods, as well as Supplementary Table 2 for detailed statistical analysis.

We then bilaterally injected the AAV9-CMV-GFP-Cre in dKO^fx/fx^ mice targeting 10 different coordinates of the cerebral cortex, which were defined based on a grid designed according to the anterior-posterior and medial-lateral axes of the mouse brain atlas (32; Fig. 3A–K, see Materials and Methods for detailed information).

6 weeks after intracerebral injection at the selected sites, we examined the presence of autistic-like behaviours in the dKO^fx/fx^ mice. Since the loss of *Shank2* and *Shank3* in the ACB leads to specific behavioural abnormalities, we used this area as a reference for our screening of cortical regions.

First, we investigated the manifestation of repetitive behaviours by comparing the effect of *Shank2/3* loss in the different cortical regions to those observed in ACB dKO^fx/fx^. We found that simultaneous deletion of the two PSD proteins in the primary somatosensory (SSp) and primary motor (MOp) area strongly increased the time spent grooming in dKO^fx/fx^ mice (Fig. 3C, L). Yet, we did not detect any repetitive behaviour by selectively targeting the SSp and the motor areas (MOp + MOs) independently and in none of the other selected regions as well (Fig. 3D, E, L). These data suggested that *Shank2/3* loss within the single subcortical areas that we investigated might lead to a different set of autistic-like features rather than to repetitive behaviours. To further test this hypothesis, we evaluated the presence of altered sociability in dKO^fx/fx^ mice after cortical injections of Cre.

In the nesting behaviour test, we could not detect any alteration after selective *Shank2/3* deletion (Fig. 3M), within the selected cortical regions. On the other hand, we found that mice lacking *Shank2/3* in the olfactory areas (OLF) showed a preference index for sociability equal to zero in the three-chamber test (Fig. 3F, N). In addition, deletion of *Shank2/3* in the retrosplenial area (RSP) triggered a loss of interest for the S2 animal in the social novelty trial of the three-chamber test (Fig. 3K, O). Of note, a similar impairment was observed also when co-targeting the posterior parietal association areas (PTLp), visual areas (VIS) and RSP (Fig. 3H, O).

Since the stereotaxic injections of AAV9-CMV-GFP-Cre in the OLF, as well as in the RSP targeted single subcortical regions, we then focus our study on these two areas.

### Retrosplenial area plays a crucial role in social memory

First, we sought confirmation of the results obtained with our cortical screening focusing on the OLF and RSP subregions. After bilateral injections of AAV9-CMV-GFP-Cre or the control virus (AAV9-CMV-GFP) in the OLF (Supplementary Fig. 2A) of dKO^fx/fx^ mice, we could not observe any altered sociability. In fact, in contrast with what was observed in the screening, GFP- and Cre-expressing mice displayed a significant preference for S1 compared to the empty cage (Supplementary Fig. 2B–E). Moreover, during the olfactory habituation/dishabituation test, there was no significant difference in the time spent sniffing the odour cues between GFP- and Cre-expressing mice (Supplementary Fig. 2F). In line with the findings on the constitutive dKO mice, this result indicates that the loss of both PSD proteins does not affect olfactory functions.

**Fig. 4:**
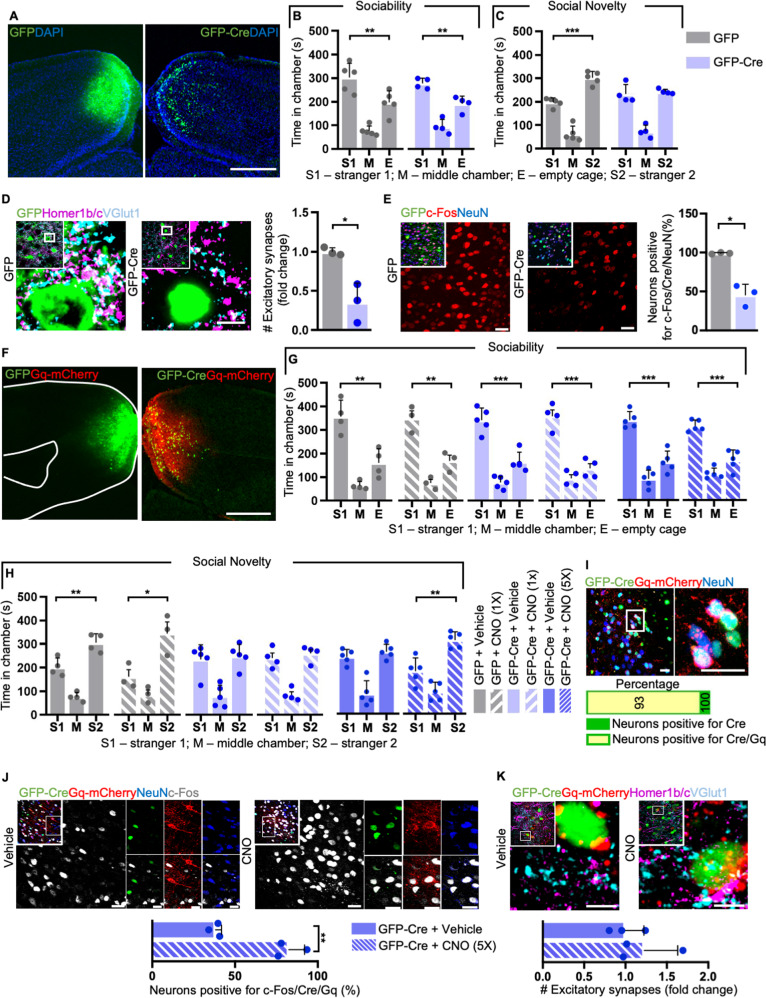
Lack of *Shank2/3* in the retrosplenial area leads to social memory deficits and excitatory synapses loss, which cannot be rescued by acute DREADD-mediated neuronal activation. **A** Representative images showing GFP (left) and GFP-Cre (right) expression in the retrosplenial area (RSP). **B** GFP- and Cre-expressing mice preferred to investigate S1 instead of the empty cage; GFP *n* = 5, GFP-Cre *n* = 4. **C** Cre-expressing mice had no preference between the S1 and the S2; GFP *n* = 5, GFP-Cre *n* = 4. **D** Immunohistochemistry (IHC) and quantification of excitatory synapses in the RSP using the Homer1b/c and VGlut1 markers; GFP *n* = 3, GFP-Cre *n* = 3. **E** Immunohistochemistry (IHC) and quantification of neurons positive for c-Fos after three-chamber test; GFP *n* = 3, GFP-Cre *n* = 3. **F** Representative images showing GFP (left) and GFP-Cre/Gq (right) expression in the RSP. **G** GFP- and Cre-expressing mice treated with either vehicle (veh) or CNO are sociable; GFP + veh *n* = 4, GFP-Cre + veh *n* = 5, GFP + CNO[1x] *n* = 3, GFP-Cre + CNO[1x] *n* = 4, GFP + CNO[5x] *n* = 5, GFP-Cre + CNO[5x] *n* = 5. **H** Cre-expressing mice treated with CNO still showed social memory deficits; GFP + veh *n* = 4, GFP-Cre + veh *n* = 5, GFP + CNO[1x] *n* = 3, GFP-Cre + CNO[1x] *n* = 4, GFP + CNO[5x] n = 5, GFP-Cre + CNO[5x] *n* = 5. **I** IHC showing that 93% of Cre-positive neurons also expressed Gq; GFP-Cre + CNO *n* = 3. **J** IHC showing that CNO administration increased c-Fos levels in the RSP; GFP-Cre + Veh[5x] *n* = 3, GFP-Cre + CNO[5x] *n* = 3. **K** IHC showing that repeated increase of neuronal firing did not rescue the loss of excitatory synapses in the RSP of Cre-expressing mice; GFP-Cre + Veh[5x] *n* = 3, GFP-Cre + CNO[5x] *n* = 3. Scale bars: 500 μm in (**A**, **F**), 5 μm in (**D**–**K**), 20 μm in (**E, I**, and **J**). See Materials and Methods, as well as Supplementary Table 2 for detailed statistical analysis.

On the other hand, we could confirm that *Shank2/3* loss within the RSP (which displays a homogeneous distribution of the two postsynaptic proteins at levels comparable to other cortical regions; Fig. 4A and Supplementary Fig. 3A, B) induces ASD-like social alterations. In fact, while during the social trial of the three-chamber test mice of both groups spent more time investigating the S1 instead of the empty cage (Fig. 4B and Supplementary Fig. 3C–E), Cre-expressing mice showed no preference between S1 and the S2 (Fig. 4C and Supplementary Fig. 3F–H). Accordingly, half of the Cre-expressing mice in the RSP exhibited a reduced preference index for the novel stimulus (Supplementary Fig. 3H).

To exclude possible off-target effects on the altered behaviour observed in dKO^fx/fx^ mice, we injected WT animals with Cre-GFP and compared their performance in the three-chambers test to non-injected littermates. These experiments did not reveal any cognitive impairment in mice expressing Cre, as they performed similarly to the control ones in both sociability and social novelty paradigms (Supplementary Fig. 4A, B). Thus, the behavioural impairment observed in the dKO^fx/fx^ animals is specifically triggered by *Shank2/3* loss within the RSP, and not by the injection and expression of the Cre recombinase per se.

We then further investigated the consequences of *Shank2/3* deletion within the RSP and found that this neither induced repetitive behaviours (Supplementary Fig. 5A) nor affected the innate nesting behaviour (Supplementary Fig. 5B). This not only confirmed our initial screening, but also highlighted a specific effect on sociability triggered by altered synaptic contacts within the RSP. Indeed, mice lacking the two PSD scaffolds displayed a significant loss of excitatory synapses detected with the independent markers VGlut1 and Homer1b/c (Fig. 4D), while the number of inhibitory VGAT-positive terminals remained unchanged upon Cre injection (Supplementary Fig. 6).

Having observed a drastic reduction of excitatory synapses in dKO^fx/fx^ mice, we asked if it might correlate with altered neuronal plasticity and recruitment. For this, we monitored the immediate-early gene c-Fos after cognitive tasks. After the three-chamber test the number of c-Fos positive neurons was significantly higher in GFP-injected mice than in CRE-expressing ones, indicating altered neuronal recruitment (Fig. 4E).

Since the RSP is known to play a crucial role in spatial navigation [41], we then performed the Barnes maze test to evaluate the learning and spatial memory abilities of our mutant animals. As GFP- and Cre-expressing mice performed similarly when searching for the escape box during the training and the probe trials (Supplementary Fig. 7A–C), these findings demonstrate that *Shank2/3* loss in the RSP impairs social memory, but not spatial memory.

We then asked whether we could rescue the social memory impairments by enhancing neuronal firing in the RSP of *Shank2/3*-deficient mice by using DREADD-based chemogenetics. To this end, we bilaterally co-injected an AAV9-CMV-GFP-Cre and an AAV9-hSyn-DIO-hM3D(Gq)-mCherry (henceforth Gq) into the RSP of dKO^fx/fx^ mice (Fig. 4F). As expected, 6 weeks post-surgery GFP- and Cre-expressing mice treated with either vehicle or a single dose [1x] of 5 mg/kg clozapine N-oxide (CNO) preferred to interact with the unfamiliar mouse S1 instead of an empty cage (Fig. 4G). However, Cre-expressing mice treated with CNO still showed no preference between S1 and S2 in the social novelty trial (Fig. 4H). This suggested that acute increase in neuronal activity is not sufficient to rescue the altered sociability triggered by *Shank2/3* loss in the RSP and that a prolonged treatment would have been required to rescue the cognitive phenotype. To test this hypothesis, we injected an independent cohort of Cre-expressing dKO^fx/fx^ mice with Gq and treated them with vehicle or CNO for 5 consecutive days [5x] prior repeating the behavioural tests. Mice of both groups performed again similarly in the sociability test but, most notably, we found that prolonged administration of CNO could rescue the social memory defects in *Shank2/3 KO* animals (Fig. 4H). Indeed, the dKO^fx/fx^ mice that received the treatment showed a significantly increased preference index for the novel stranger animal than for the familiar one, indicating a restoration of cognitive features involved in social behaviours through neuronal stimulation (Supplementary Fig. 8).

To confirm this hypothesis, we evaluated the neuronal recruitment upon DREADD activation and observed that 93% of the Cre-positive neurons also expressed the Gq-receptor (Fig. 4I). Moreover, administration of CNO in Cre-expressing dKO^fx/fx^ mice significantly increased the number of c-Fos/Cre/Gq-positive neurons in comparison to vehicle-treated animals (Fig. 4J), thus confirming DREADD activation and, importantly, suggesting an improved neuronal recruitment upon cognitive tasks. Notably, despite showing signs of increased plasticity (as shown by the c-Fos levels), Gq activation was not sufficient to rescue the number of excitatory synapses within the RSP of Cre-expressing mice (Fig. 4K). All in all, our data indicate that the loss of *Shank2* and *Shank3* in the RSP leads to a dramatic synaptic disruption and altered social behaviour, that can be rescued by repeatedly increasing neuronal activity and plasticity.

## Discussion

**Fig. 5:**
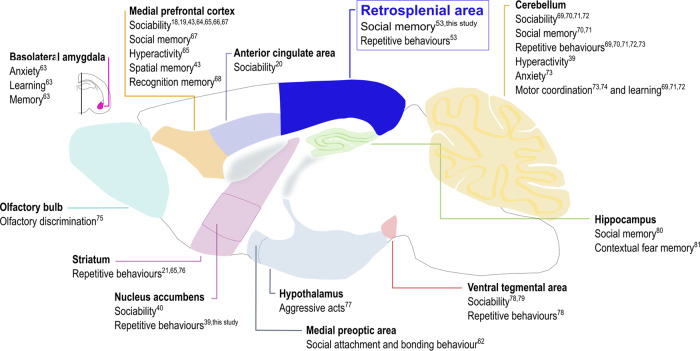
Single brain regions causally linked to ASD-related behaviours. In this schematic overview, identified subregions of the brain are highlighted and associated behavioural alterations seen in ASD models are listed [63–81].

Synaptic abnormalities and cortical dysfunction are pathological hallmarks of several neuropsychiatric and neurodevelopmental conditions, including autism spectrum disorders [3, 42]. Indeed, analysis of postmortem ASD brains revealed a pattern of structural changes affecting many cortical areas such as the prefrontal cortex, fusiform gyrus, frontoinsular cortex and cingulate cortex [9]. Furthermore, different ASD mouse models display altered and/or dysfunctional cortical subregions [11, 20, 21, 43]. For example, NMDA receptor dysfunction in the medial prefrontal cortex underlies cognitive and social impairments in *16p11*^*-/-*^ mice [43]. On the other hand, loss of dendritic spines and hypoactivity in the pyramidal neurons of the anterior cingulate area play a causal role in the social deficits observed in *Shank3* mutant mice [20]. In addition to the cerebral cortex, other brain regions such as cerebellum, hippocampus, amygdala and striatum are discussed as being important for the ASD phenotype ([9, 44]; Fig. 5). In this respect, the nucleus accumbens (a striatal subregion) has been associated with reward-related behaviours, a process affected in ASD [45], as well as with repetitive behaviours [39] and social deficits [40]. Likewise, here we show that local deletion of *Shank2 and Shank3* in the ACB triggers repetitive behaviours and a mild social novelty impairment.

Despite the significant advances in the understanding of a causal relationship between brain structures and behaviour, there are still potential unknown circuitries underlying ASDs. Thus, we used a novel screening approach based on local deletion of *Shank2* and *Shank3* genes in different coordinates of the cortex. Altered synaptic contacts are observed in ASD patients and animal models [9]. In particular: increased dendritic spine density was observed in *Fmr1*^−/−^- and *Tsc2*^*+/*−^- ASD mice [46, 47], while reduced number of spines was reported in *Mecp2*- and *Shank3*- deficient animals [48, 49]. We decided to delete two high-confidence candidate ASD genes that encode for synaptic proteins because previous studies demonstrated that mice lacking multiple synaptic proteins display aggravated abnormalities at the synapses [50, 51]. Consistently, simultaneous loss of both postsynaptic proteins induced a significant loss of excitatory synapses and impaired neuronal recruitment and plasticity within the retrosplenial area. Importantly, despite the *Shank2/3* deletion was not specifically carried out in excitatory neurons, it did not appear to affect the number of presynaptic inhibitory terminals, indicating the presence of synaptic excitation/inhibition imbalance in our model [52].

In addition to the loss of excitatory synapses in the RSP, we observed that *Shank2/3* loss in this cortical region results in social memory deficits, without triggering other ADS-related phenotypes. Interestingly, the RSP has been associated not only to social memory impairments, but also to repetitive behaviours in a constitutive, neurodevelopmental *Senp1*-deficient mouse model [53]. Therefore, early changes in development and plasticity in the retrosplenial area might trigger a broader spectrum of behavioural phenotypes, which might even involve cells other than neurons. In fact, glial dynamics within the RSP have been linked to memory performance [54].

Given the connectivity of the RSP with the hippocampus and the anterior thalamic nuclei, this cortical area is known to be crucial in navigation and spatial memory [41]. However, we observed that mice lacking *Shank2/3* in the RSP exhibited normal spatial learning ability, indicating that the local genetic insult specifically affects social memory, and not spatial memory. The retrosplenial area is found to be involved not only in spatial memory tasks, but also when the brain is in a resting state: the so-called default mode network (DMN; [55]). The DMN comprises several brain regions, including the RSP, and is typically activated when an individual is not performing any task [56]. However, some brain areas belonging to this network are often engaged during social tasks [57]. Interestingly, hyperconnectivity of DMN was previously linked to the social deficits in children with ASD [58] and altered DMN connectivity was also observed in a *Shank3* KO mouse model [59]. Given the overlap between the DMN and brain regions involved in sociability, we can hypothesize that the retrosplenial area may be involved in social cognition.

Although autism is a developmental disorder, previous studies demonstrated that autistic-like behaviours can be triggered [39] or even rescued after brain development [60]. For example, Guo and colleagues [20] showed that acute activity enhancement within the anterior cingulate area of adult *Shank3* KO mice improved social interaction deficits. However, we observed that a single DREADD-mediated neuronal stimulation within the RSP after local deletion of *Shank2* and *Shank3* is not sufficient to rescue the social memory impairments. One possible explanation may be the time-dependent loss of the scaffold proteins. While Guo and collaborators [20] performed the rescue experiments in a mouse model which underwent brain development after the genetic insult, our rescue experiments were performed in a context where *Shank2*/*3* deletion occurred after the physiological neuronal development [22]. On the other hand, simultaneous loss of both Shank2 and Shank3 proteins may prevent a possible compensatory mechanism at glutamatergic synapses of the single scaffold proteins when only one is missing. The constitutive loss of *Shank1* and *Shank3* genes, which leads to dramatic synaptic defects and autistic-like behaviours, can indeed be rescued upon pharmacological re-establishment of spine density [61]. Nevertheless, repeated chemogenetic activation of the medial preoptic area can restore maternal behaviour in *Shank2* KO mice [62], suggesting that the neurotrophic effect of prolonged activity can bypass the loss of synaptic contacts. In line with this, prolonged neuronal stimulation rescued the social memory impairments even without elevating the number of synapses of dKO^fx/fx^ mice. Thus, despite the aggravated loss of excitatory synapses in the RSP appears as a structural determinant of the cognitive alteration observed in *Shank2/3*-deficient animals, this can be overcome by interventions at the level of neuronal plasticity.

## Supplementary information

Suppl Figures and legends

Suppl Tables

Suppl Video 1

Suppl Video 2
