# Supplementary material for: Shank2/3 double knockout-based screening of cortical subregions links the retrosplenial area to the loss of social memory in autism spectrum disorders
Source: Mol Psychiatry. 2022 Sep 13;27(12):4994–5006. doi: 10.1038/s41380-022-01756-8 (PMC9763120; doi:10.1038/s41380-022-01756-8)
Supplement: Supplementary file 1 — Suppl Figures and legends [file 41380_2022_1756_MOESM1_ESM.docx]

***Shank2/3* double knockout-based screening of cortical subregions links the retrosplenial area to the loss of social memory in autism spectrum disorders**

Débora Garrido (MSc.)^1,2, #^, Stefania Beretta (Dr.)^3, #^, Stefanie Grabrucker (Dr.)^1^, Helen Friedericke Bauer (MSc.)^1,2^, David Bayer (Dr.)^2,4^, Carlo Sala (Prof. Dr.)^5^, Chiara Verpelli (Dr.)^5^, Francesco Roselli (Prof. Dr.)^3,4^, Juergen Bockmann (Dr.)^1^, Christian Proepper (Dr.)^1^, Alberto Catanese (Dr.)^1,3^, Tobias M. Boeckers (Prof. Dr.)^1,3^

1 Institute of Anatomy and Cell Biology, Ulm University, 89081 Ulm, Germany

2 International Graduate School, Ulm University, 89081 Ulm, Germany

3 German Center for Neurodegenerative Diseases (DZNE), Ulm site, 89081 Ulm, Germany

4 Department of Neurology, Ulm University, 89081 Ulm, Germany

5 CNR, Institute for Neuroscience, Milano, Italy

# co-first authors

**Supplementary information:** contains Supplementary Figures 1-8, as well as Supplementary Video Legends 1 and 2

**Supplementary Figure 1**

**
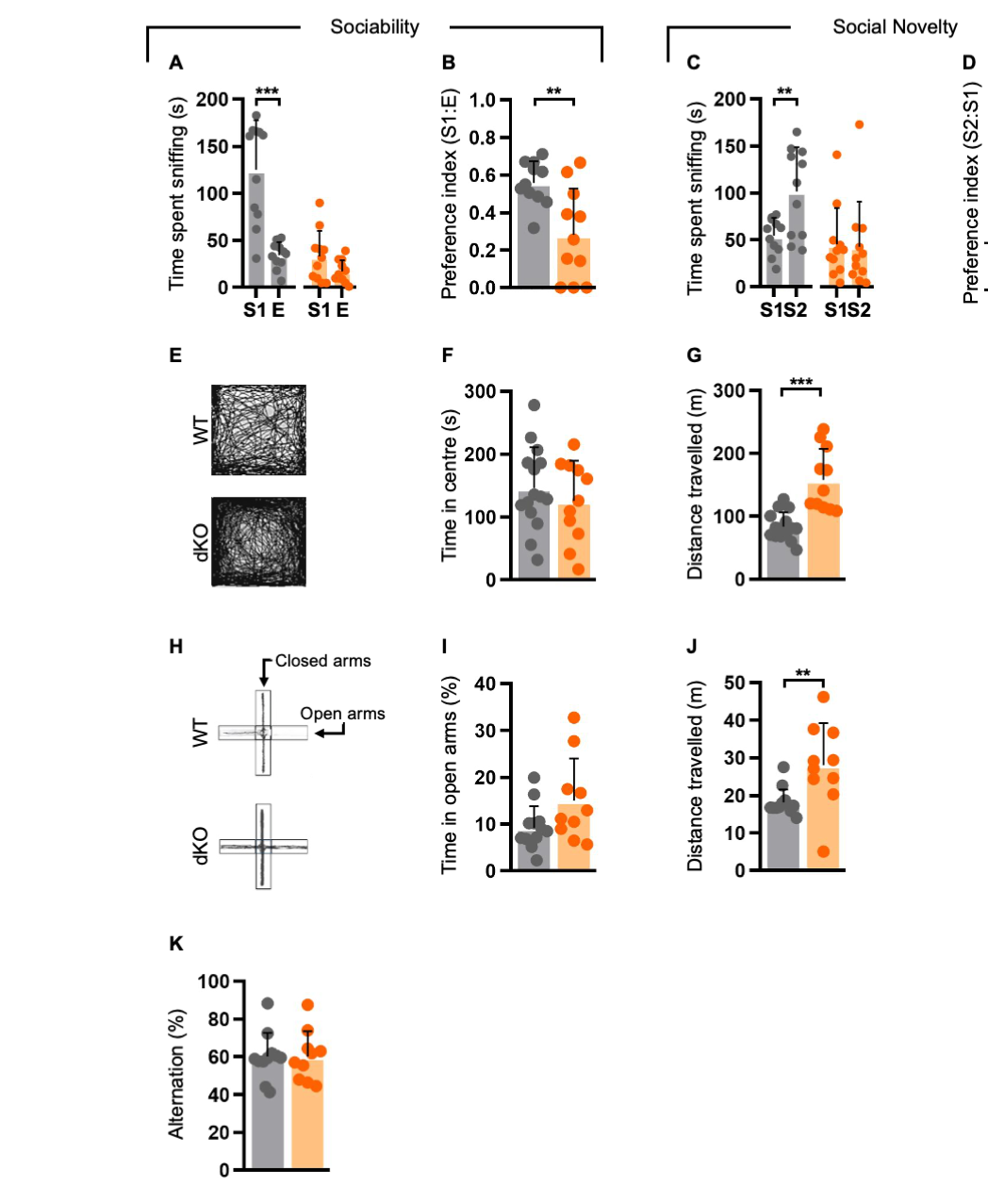
**

**Supplementary Figure 1: Social impairments and hyperactivity in dKO mice. A-B**, dKO mice had no preference between S1 and an empty cage (**A**) and showed reduced social preference index (**B**) in comparison to WT animals; WT = 11, dKO = 11. C-D, While WT mice spent more time in close interaction with the S2, dKO displayed no preference between S1 and S2 (**C**). Moreover, a large number of dKO mice showed reduced preference index for the novel stimulus (**D**); WT = 11, dKO = 11. E-G, Representative tracks of WT and dKO animals in the open field arena (**E**). No significant differences were detected in the time spent in the centre zone between WT and dKO mice (**F**), but the distance travelled by the dKO animals was significantly increased in comparison to the WT mice (**G**), suggesting that dKO mice are hyperactive; WT = 15, dKO = 11. H-J, Examples of trajectories of WT and dKO animals in the elevated plus maze (**H**). dKO mice did not display anxiety-like behaviour as no significant differences were detected in time spent in open arms between WT and mutant mice (**I**), however dKO mice travelled a longer distance in comparison to control mice (**J**); WT = 13, dKO = 10. K, dKO mice displayed no impairments in spontaneous alternation behaviour in the Y maze arena; WT = 11, dKO = 10. See Materials and Methods, as well as Supplementary Table 2 for detailed statistical analysis.

**Supplementary Figure 2**

**
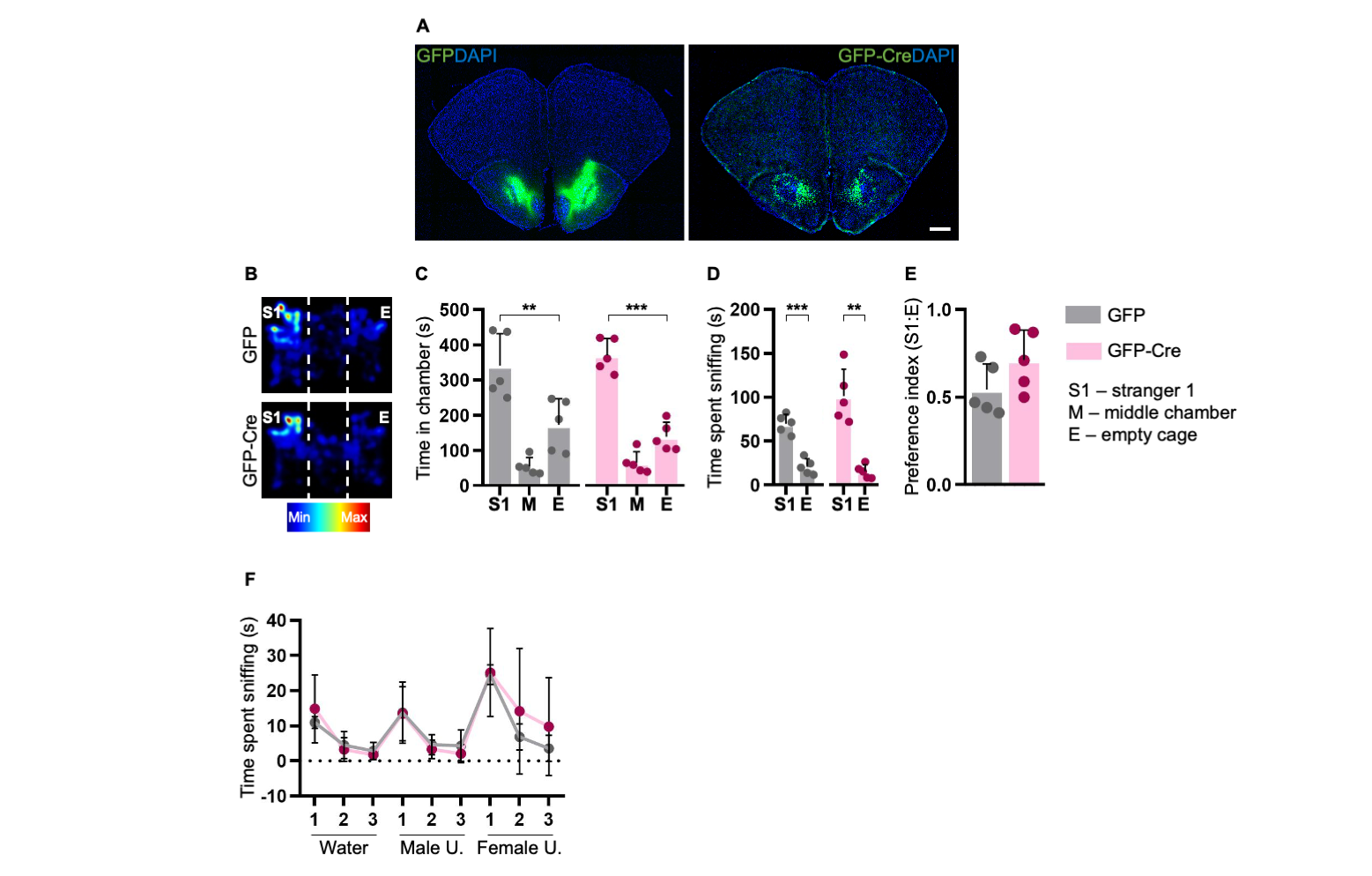
**

**Supplementary Figure 2: Shank2/3 loss in the olfactory areas neither affects sociability nor olfactory functions. A**, Representative images showing expression of GFP (left) and GFP-Cre (right) in the olfactory areas (OLF). **B**, Representative heatmaps of the three-chamber sociability trial. **C-E**, GFP- and Cre-expressing mice preferred to investigate S1 instead of the empty cage; GFP n = 5, GFP-Cre n = 5. **F**, No significant differences were observed in the time spent sniffing the odour cues between GFP- and Cre-expressing animals; GFP n = 5, GFP-Cre n = 5. Male U., male urine; Female U., female urine. Scale bar: 500 μm. See Materials and Methods, as well as Supplementary Table 2 for detailed statistical analysis.

**Supplementary Figure 3**

**
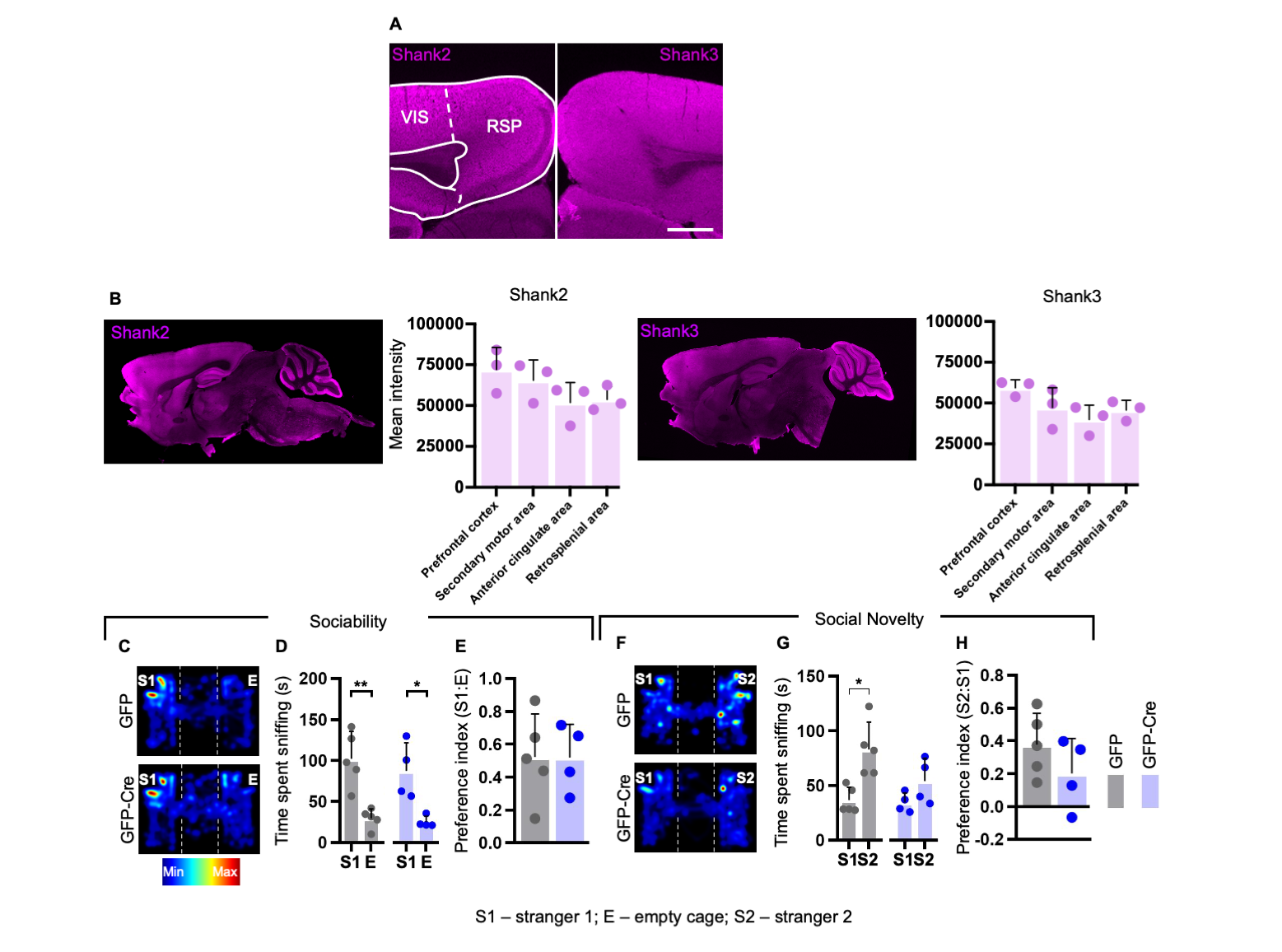
**

**Supplementary Figure 3: Shank2 and Shank3 proteins are homogenously expressed in the retrosplenial area and loss of these two synaptic proteins in this cortical region affects social memory. A**, Representative images showing expression of Shank2 (left) and Shank3 (right) in the RSP. VIS, visual areas; RSP, retrosplenial area. **B**, Immunohistochemistry and quantification of Shank2 and Shank3 distribution in cortical subregions; n = 3. **C**, Representative heatmaps of the three-chamber sociability trial. **D-E**, GFP- and Cre-expressing mice in the RSP spent more time investigating the S1 instead of an empty cage (**D**) and the preference index for sociability was comparable between both groups (**E**); GFP n = 5, GFP-Cre n = 4. **F**, Representative heatmaps of the three-chamber social novelty trial. **G-H**, Cre-expressing mice within the RSP displayed social memory deficits, as they showed no preference between S1 and S2 (**G**) and half of the Cre-expressing mice exhibited a reduced preference index for the novel stimulus (**H**); GFP n = 5, GFP-Cre n = 4. Scale bar: 500 μm. See Materials and Methods, as well as Supplementary Table 2 for detailed statistical analysis.

**Supplementary Figure 4**


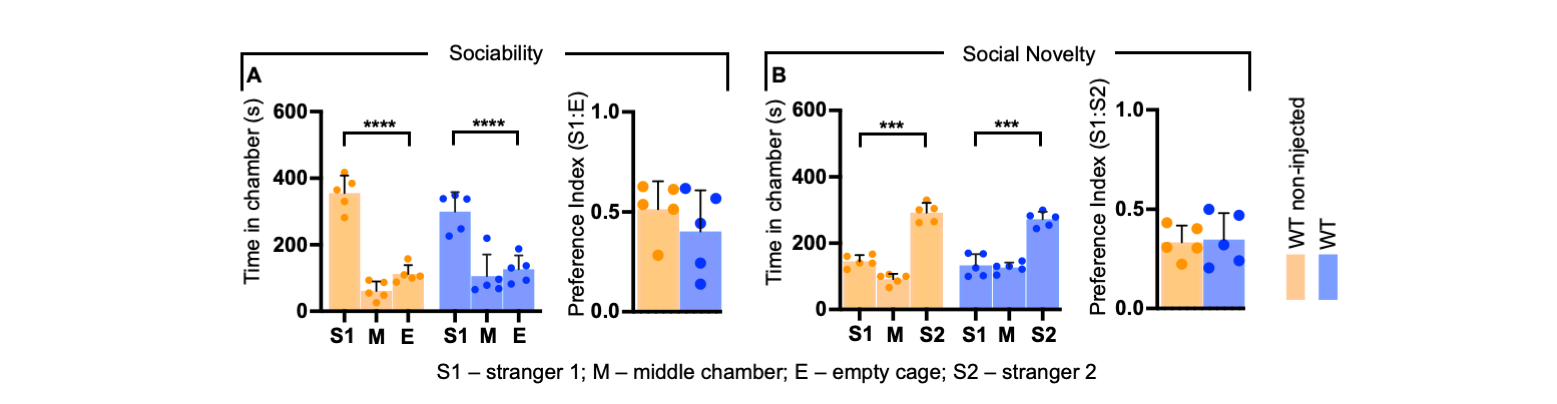


**Supplementary Figure 4: Cre-recombinase does not alter the behavior of WT mice. A-B**, Cre-expressing WT animals perform similarly to non-injected ones in the sociability (**A**) and social novelty (**B**) tasks; non-injected n = 3, GFP-Cre n = 3.

**Supplementary Figure 5**

**
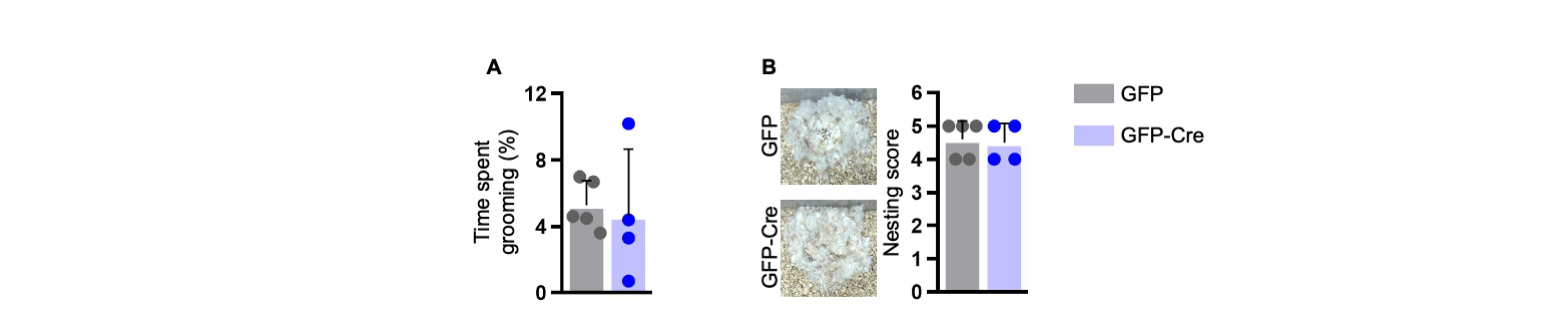
**

**Supplementary Figure 5: Shank2/3 loss within the RSP does not induce repetitive behaviours. A-B,** quantification of the time spent self-grooming (**A**) and nest building behaviour (**B**) in dKO^fx/fx^; GFP n = 5, GFP-Cre n = 4.

**Supplementary Figure 6**

**
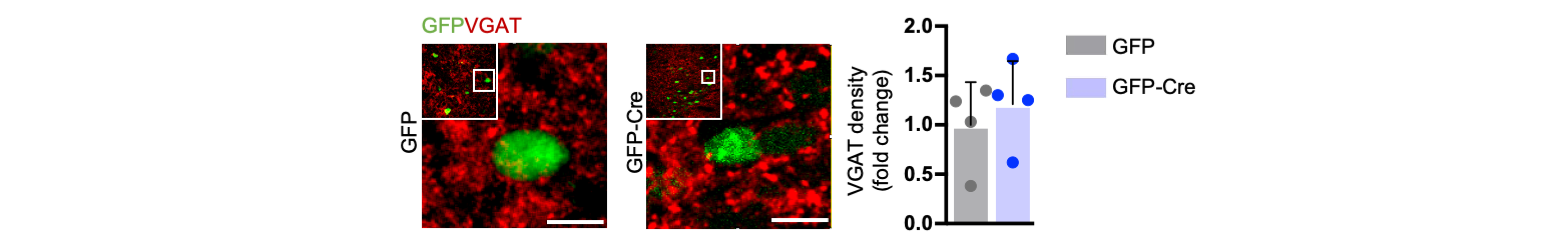
**

**Supplementary Figure 6: deletion of Shank2 and Shank3 does not affect the number of inhibitory synapses.** Immunohistochemistry and quantification of inhibitory pre-synaptic terminals stained against VGAT in the RSP of GFP- and Cre-injected mice; GFP n = 3, GFP-Cre n = 3. Scale bar: 10 μm.

**Supplementary Figure 7**


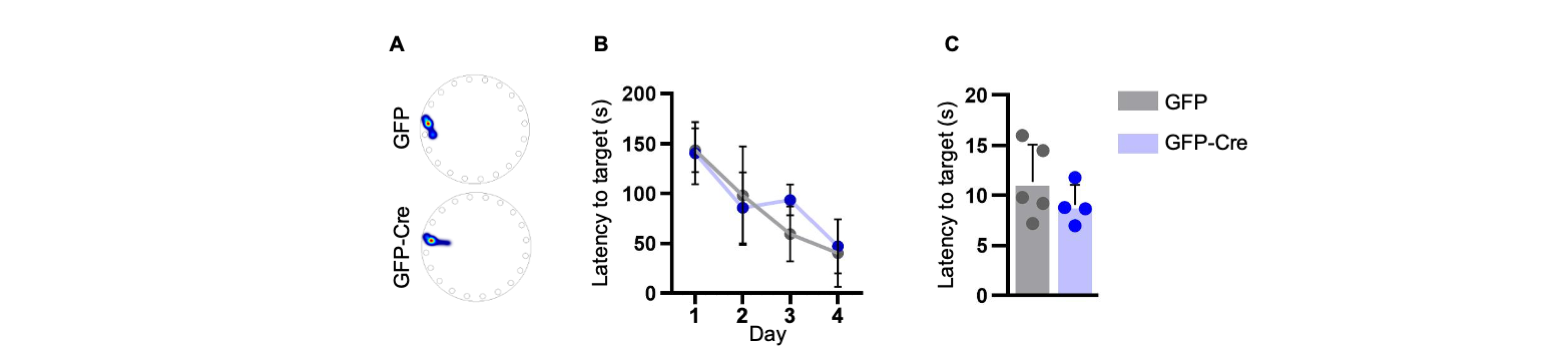


**Supplementary Figure 7: spatial navigation is not altered by the loss of the two scaffold proteins. A,** Representative heatmaps of the Barnes maze probe trials. **B-C,** Training (**B**) and probe (**C**) trials of the Barnes maze test; GFP n = 5, GFP-Cre n = 4.

**Supplementary Figure 8**

**
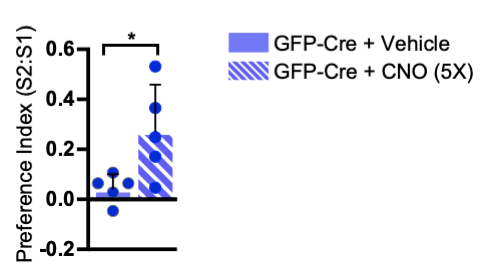
**

**Supplementary Figure 8: prolonged neuronal activation in the RSP ameliorates social memory upon Shank2/3 deficiency.** Mice repeatedly treated with CNO have higher preference for the novel stranger animal than vehicle-injected ones; GFP-Cre + Veh(5x) n = 3, GFP-Cre + CNO(5x) n = 3.

**Supplementary Video legends**

**Supplementary Video 1:** This video shows an example of social behaviour deficits in a constitutive dKO mouse during reciprocal interaction with a WT mouse.

**Supplementary Video 2:** This video shows an example of repetitive jumping behaviour mixed with upright scrabbling acts in a constitutive dKO mouse.
