# Supplementary material for: Shank2/3 double knockout-based screening of cortical subregions links the retrosplenial area to the loss of social memory in autism spectrum disorders
Source: Mol Psychiatry. 2022 Sep 13;27(12):4994–5006. doi: 10.1038/s41380-022-01756-8 (PMC9763120; doi:10.1038/s41380-022-01756-8)
Supplement: Supplementary file 2 — Suppl Tables [file 41380_2022_1756_MOESM2_ESM.docx]

***Shank2/3* double knockout-based screening of cortical subregions links the retrosplenial area to the loss of social memory in autism spectrum disorders**

Débora Garrido (MSc.)^1,2, #^, Stefania Beretta (Dr.)^3, #^, Stefanie Grabrucker (Dr.)^1^, Helen Friedericke Bauer (MSc.)^1,2^, David Bayer (Dr.)^2,4^, Carlo Sala (Prof. Dr.)^5^, Chiara Verpelli (Dr.)^5^, Francesco Roselli (Prof. Dr.)^3,4^, Juergen Bockmann (Dr.)^1^, Christian Proepper (Dr.)^1^, Alberto Catanese (Dr.)^1,3^, Tobias M. Boeckers (Prof. Dr.)^1,3^

1 Institute of Anatomy and Cell Biology, Ulm University, 89081 Ulm, Germany

2 International Graduate School, Ulm University, 89081 Ulm, Germany

3 German Center for Neurodegenerative Diseases (DZNE), Ulm site, 89081 Ulm, Germany

4 Department of Neurology, Ulm University, 89081 Ulm, Germany

5 CNR, Institute for Neuroscience, Milano, Italy

# co-first authors

**Supplementary information:** contains Supplementary Table 1 and 2

|  | **Coordinates** | **Targeted brain regions** |
| --- | --- | --- |
|  | AP + 1.7; ML ± 0.75 ; DV -3.75 | Nucleus accumbens (ACB) |
|  | AP + 1; ML ± 0.3; DV - 1.1 | Anterior cingulate area (ACA) and secondary motor area (MOs) |
|  | AP - 1; ML ± 2; DV - 0.6 | Primary somatosensory area (SSp) and primary motor area (MOp) |
|  | AP - 1; ML ± 3; DV - 0.5 | Primary somatosensory area (SSp) |
|  | AP 0; ML ± 1; DV - 0.5 | Primary motor area (MOp) and secondary motor area (MOs) |
|  | AP + 2.5; ML ± 0.9; DV – 3 | Olfactory areas (OLF) |
|  | AP - 3; ML ± 2.5; DV - 0.6 | Visual areas (VIS) |
|  | AP - 2; ML ± 1.5; DV - 0.5 | Posterior parietal association areas (PTLp), visual areas (VIS) and retrosplenial area (RSP) |
|  | AP - 2; ML ± 3; DV - 0.6 | Primary somatosensory area (SSp), visual areas (VIS) and posterior parietal association areas (PTLp) |
|  | AP + 2; ML ± 1; DV - 0.6 | Secondary motor area (MOs) |
|  | AP - 3; ML ± 0.3; DV - 0.5 | Retrosplenial area (RSP) |

**Supplementary Table 1: Stereotaxic coordinates used.**

AP, anterior-posterior; ML, medial-lateral; DV, dorsal-ventral. All numbers are in millimetres.

**Supplementary Table 2: Statistical information for figures and supplementary figures.**

| Figure | Panel (group) | Statistical test | p-value |
| --- | --- | --- | --- |
| 1 | B (WT *vs* dKO) | Mann–Whitney test | <0.0001 |
|  | C (WT *vs* dKO) | Mann–Whitney test | <0.0001 |
|  | D (WT) | Kruskal-Wallis test with Dunn’s post hoc test | 0.0229 |
|  | D (dKO) | One-way ANOVA with Tukey’s post hoc test | 0.6783 |
|  | E (WT) | One-way ANOVA with Tukey’s post hoc test | 0.0224 |
|  | E (dKO) | Kruskal-Wallis test with Dunn’s post hoc test | 0.3682 |
|  | G (WT *vs* dKO) | Mann–Whitney test | 0.0014 |
|  | H (WT *vs* dKO) | Mann–Whitney test | 0.0002 |
|  | I (WT *vs* dKO) | Mann–Whitney test | 0.0003 |
|  | J (WT *vs* dKO) | Mann–Whitney test | 0.0070 |
|  | K (WT *vs* dKO) | Welch‘s t test | 0.4976 |
|  | M (WT *vs* dKO) | Mann–Whitney test | 0.0090 |
|  | N (WT *vs* dKO) | Mann–Whitney test | 0.5569 |
|  | O (WT *vs* dKO) | Chi-square test | <0.0001 |
|  | P (WT *vs* dKO) | Mann–Whitney test | 0.0638 |
|  | Q (WT *vs* dKO) | Chi-square test | <0.0001 |
| 2 | C (GFP *vs* GFP-Cre) | Welch‘s t test | 0.0024 |
|  | D (GFP *vs* GFP-Cre) | Welch‘s t test | 0.0301 |
|  | E (GFP *vs* GFP-Cre) | Mann–Whitney test | 0.0043 |
|  | F (GFP *vs* GFP-Cre) | Mann–Whitney test | 0.1905 |
|  | H (GFP) | One-way ANOVA with Tukey’s post hoc test | <0.0001 |
|  | H (GFP-Cre) | Kruskal-Wallis test with Dunn’s post hoc test | 0.0283 |
|  | I (GFP) | Mann–Whitney test | 0.0079 |
|  | I (GFP-Cre) | Mann–Whitney test | 0.0022 |
|  | J (GFP *vs* GFP-Cre) | Mann–Whitney test | 0.7597 |
|  | L (GFP) | One-way ANOVA with Tukey’s post hoc test | 0.0019 |
|  | L (GFP-Cre) | One-way ANOVA with Tukey’s post hoc test | <0.0001 |
|  | M (GFP) | Mann–Whitney test | 0.0079 |
|  | M (GFP-Cre) | Welch‘s t test | 0.0146 |
|  | N (GFP *vs* GFP-Cre) | Welch‘s t test | 0.0108 |
| 3 | L [(ACB (GFP) *vs* ACB (GFP-Cre)] | Mann–Whitney test | 0.0043 |
|  | M [(ACB (GFP) *vs* ACB (GFP-Cre)] | Mann–Whitney test | 0.1905 |
|  | N [(ACB (GFP) *vs* ACB (GFP-Cre)] | Mann–Whitney test | 0.7597 |
|  | O [(ACB (GFP) *vs* ACB (GFP-Cre)] | Welch‘s t test | 0.0108 |
| 4 | B (GFP) | One-way ANOVA with Tukey’s post hoc test | 0.0095 |
|  | B (GFP-Cre) | One-way ANOVA with Tukey’s post hoc test | 0.0052 |
|  | C (GFP) | One-way ANOVA with Tukey’s post hoc test | 0.0001 |
|  | C (GFP-Cre) | One-way ANOVA with Tukey’s post hoc test | 0.8862 |
|  | D (GFP *vs* GFP-Cre) | Welch‘s t test | 0.0408 |
|  | E (GFP *vs* GFP-Cre) | Welch‘s t test | 0,0207 |
|  | G (GFP + veh) | One-way ANOVA with Tukey’s post hoc test | 0.0015 |
|  | G (GFP + CNO) (1x) | One-way ANOVA with Tukey’s post hoc test | 0.0054 |
|  | G (GFP-Cre + veh) | One-way ANOVA with Tukey’s post hoc test | <0.0001 |
|  | G (GFP-Cre + CNO) (1x) | One-way ANOVA with Tukey’s post hoc test | 0.0001 |
|  | G (GFP-Cre + veh) | One-way ANOVA with Tukey’s post hoc test | <0.0001 |
|  | G (GFP-Cre + CNO) (5x) | One-way ANOVA with Tukey’s post hoc test | <0.0001 |
|  | H (GFP + veh) | One-way ANOVA with Tukey’s post hoc test | 0.0042 |
|  | H (GFP + CNO) (1x) | One-way ANOVA with Tukey’s post hoc test | 0.0238 |
|  | H (GFP-Cre + veh) | Kruskal-Wallis test with Dunn’s post hoc test | >0.9999 |
|  | H (GFP-Cre + CNO) (1x) | One-way ANOVA with Tukey’s post hoc test | 0.7235 |
|  | H (GFP-Cre +veh) | One-way ANOVA with Tukey’s post hoc test | 0.6727 |
|  | H (GFP-Cre + CNO) (5x) | One-way ANOVA with Tukey’s post hoc test | 0.0012 |
|  | J (GFP-Cre + veh *vs* GFP-Cre + CNO) (5x) | Welch‘s t test | 0.0083 |
|  | K (GFP-Cre + veh *vs* GFP-Cre + CNO) (5x) | Welch‘s t test | 0.4538 |
| SF1 | A (WT) | Welch‘s t test | 0.0002 |
|  | A (dKO) | Welch‘s t test | 0.0905 |
|  | B (WT *vs* dKO) | Welch‘s t test | 0.0044 |
|  | C (WT) | Welch‘s t test | 0.0087 |
|  | C (dKO) | Mann–Whitney test | 0.6411 |
|  | D (WT *vs* dKO) | Welch‘s t test | 0.0643 |
|  | F (WT *vs* dKO) | Welch‘s t test | 0.4393 |
|  | G (WT *vs* dKO) | Welch‘s t test | 0.0004 |
|  | I (WT *vs* dKO) | Welch‘s t test | 0.0825 |
|  | J (WT *vs* dKO) | Mann–Whitney test | 0.0025 |
|  | K (WT *vs* dKO) | Welch‘s t test | 0.9867 |
| SF2 | C (GFP) | One-way ANOVA with Tukey’s post hoc test | 0.0064 |
|  | C (GFP-Cre) | One-way ANOVA with Tukey’s post hoc test | <0.0001 |
|  | D (GFP) | Welch‘s t test | <0.0001 |
|  | D (GFP-Cre) | Welch‘s t test | 0.0025 |
|  | E (GFP *vs* GFP-Cre) | Welch‘s t test | 0.1334 |
|  | F (GFP *vs* GFP-Cre) | Two-way ANOVA with Sidak's post hoc test | 1: 0.9912  2:>0.9999  3:>0.9999  1:>0.9999  2:>0.9999  3: 0.9999  1:>0.9999  2: 0.7089  3: 0.8585 |
| SF3 | B (Shank2) | One-way ANOVA with Tukey’s post hoc test | 0.1868 |
|  | B (Shank3) | One-way ANOVA with Tukey’s post hoc test |  |
|  | D (GFP) | Welch‘s t test | 0.0056 |
|  | D (GFP-Cre) | Mann–Whitney test | 0.0286 |
|  | E (GFP *vs* GFP-Cre) | Welch‘s t test | 0.9844 |
|  | G (GFP) | Welch‘s t test | 0.0102 |
|  | G (GFP-Cre) | Welch‘s t test | 0.1521 |
|  | H (GFP *vs* GFP-Cre) | Welch‘s t test | 0.2447 |
| SF4 | A (WT non-injected) | One-way ANOVA with Tukey’s post hoc test | <0.0001 |
|  | A (WT GFP-Cre) | One-way ANOVA with Tukey’s post hoc test | 0.0001 |
|  | A (WT non-injected vs WT GFP-Cre) | Welch‘s t test | 0.3442 |
|  | B (WT non-injected) | One-way ANOVA with Tukey’s post hoc test | <0.0001 |
|  | B (WT GFP-Cre) | One-way ANOVA with Tukey’s post hoc test | <0.0001 |
|  | B (WT non-injected vs WT GFP-Cre) | Welch‘s t test | 0.8512 |
| SF5 | A (GFP *vs* GFP-Cre) | Welch‘s t test | 0.7818 |
|  | B (GFP *vs* GFP-Cre) | Mann–Whitney test | >0.9999 |
| SF6 | (GFP *vs* GFP-Cre) | Welch‘s t test | 0.5202 |
| SF7 | B (GFP *vs* GFP-Cre) | Two-way ANOVA with Sidak's post hoc test | Day 1: 0.9997  Day 2: 0.9895  Day 3: 0.1980  Day 4:  0.9963 |
|  | C (GFP *vs* GFP-Cre) | Welch‘s t test | 0.2860 |
| SF8 | (GFP-Cre veh *vs* GFP-Cre CNO) (5x) | Welch‘s t test | 0.0482 |
